# Incident Atrial Fibrillation Following Anthracycline-Based Adjuvant Chemotherapy in Women with Breast Cancer: A Single-Centre Retrospective Cohort Study

**DOI:** 10.3390/biomedicines14102214

**Published:** 2026-09-30

**Authors:** Abdulla Arslan, Özgür Çağaç, Fatih Beşiroğlu, Burak Hünük

**Affiliations:** Baskent University Hospital, Istanbul 34662, Türkiye; ozgurcagac@yahoo.com (Ö.Ç.); fbesiroglu@hotmail.com (F.B.); burakhunuk@hotmail.com (B.H.)

**Keywords:** atrial fibrillation, anthracyclines, breast carcinoma, cardiotoxicity, chemotherapy

## Abstract

**Background/Objectives**: Anthracyclines carry considerable cardiotoxicity risk, and atrial fibrillation (AF) during cancer treatment adversely affects morbidity and mortality. We compared the incidence of clinically documented AF and explored its early clinical correlates between anthracycline-containing and non-anthracycline adjuvant regimens in triple-negative breast cancer (TNBC). **Methods**: This single-centre retrospective cohort included consecutive women with stage II TNBC treated with breast-conserving surgery and adjuvant doxorubicin–cyclophosphamide followed by docetaxel (AC-T) or docetaxel–cyclophosphamide (TC) between 2018 and 2023. ECG and echocardiography were performed at baseline, month one, and month six. Clinically documented AF was defined as AF documented on a scheduled or symptom-triggered 12-lead ECG or as AF lasting ≥30 s on symptom-triggered 72 h Holter monitoring. Patients with structural or coronary heart disease, complete bundle branch block, or non-sinus rhythm were excluded. **Results**: Among 140 women (mean age, 64 ± 12 years; 64 AC-T), 17 (12.1%) developed clinically documented AF within six months. AF was more frequent with AC-T (18.8% vs. 6.6%; absolute risk difference, 12.2% [95% CI, 1.1–23.3%]; risk ratio, 2.85 [1.07–7.58]; log-rank *p* = 0.030), and the association persisted after inverse probability of treatment weighting (OR, 3.28 [1.05–10.24]). At one month, PR prolongation > 20 ms was associated with incident AF (OR, 5.16 [1.79–14.89]), whereas conventional echocardiographic changes did not differ between regimens. **Conclusions**: The AC-T regimen was associated with more clinically documented incident AF than TC. Early PR prolongation, occurring without detectable changes in conventional echocardiographic parameters, may be an informative early electrical signal of AF risk, although prospective validation is required.

## 1. Introduction

Atrial fibrillation (AF) is the most prevalent cardiac arrhythmia in adults with a significant impact on mortality and morbidity and an ever-growing prevalence [1]. Women are found to have both a lower incidence and lifetime risk of AF, but are more frequently symptomatic and more likely to present with paroxysmal forms of AF at the time of incident AF diagnosis in large registries, compared with men [2,3].

Triple-negative breast cancer (TNBC) is an aggressive invasive breast carcinoma phenotype accounting for around 15% of all breast carcinomas [4]. Recurrence rates within the first three to five postoperative years as well as the success of established hormonal or HER2-targeted therapies, are currently unfavourable; therefore, cytotoxic chemotherapy appears to be the mainstay of systemic treatment [5]. Currently, anthracycline-containing protocols such as doxorubicin/cyclophosphamide followed by a taxane (AC-T) and non-anthracycline regimens such as docetaxel/cyclophosphamide (TC) in the early adjuvant setting represent the established therapeutic options [6].

Anthracycline group chemotherapeutics have previously been associated with dose-dependent left ventricular dysfunction and overt heart failure [7]. However, in recent years, a growing body of evidence has also implicated their effects in supraventricular arrhythmogenesis, most notably new-onset atrial fibrillation (AF) [8,9,10]. AF incidence is reported to range from 2% to 16% depending on diagnostic methodology, age, and baseline cardiac substrate during anthracycline therapy, and AF is also associated with early chemotherapy cessation, increased thromboembolic risk, as well as adverse cardiac outcomes [11,12]. Suggested mechanisms include direct atrial myocyte injury via topoisomerase IIβ inhibition, inflammatory/oxidative stress during the disease course and after surgery, disordered calcium handling, and altered modulation of multiple ion channels affecting both conduction and repolarization [12,13].

Studies comparing the arrhythmogenic effects of anthracycline versus non-anthracycline-based adjuvant regimens without the probable confounding effects of targeted therapies in breast cancer are scarce, and populations are heterogeneous [14,15,16]. Therefore, we conducted a single-centre retrospective cohort study to compare the incidence and clinical predictors of new onset AF between AC-T and TC regimens in a well-defined, relatively homogenous population of women with stage II TNBC undergoing adjuvant chemotherapy after breast-conserving surgery and with a low to moderate Heart Failure Association-International Cardio-Oncology Society (HFA-ICOS) baseline cardiovascular toxicity risk class [12].

## 2. Materials and Methods

### 2.1. Study Population and Design

Adult women (≥18 years) with histologically confirmed, newly diagnosed TNBC were identified between 1 January 2018 and 31 December 2023 through the institutional electronic medical records of our tertiary centre and consecutively evaluated for eligibility. TNBC was defined as oestrogen and progesterone receptor expression <1% and human epidermal growth factor receptor 2 (HER2) negativity. Eligible patients had American Joint Committee on Cancer (AJCC) 8th edition [17] pathological stage II (IIA or IIB) disease and had undergone breast-conserving surgery (lumpectomy with sentinel lymph node biopsy or axillary lymph node dissection) with histologically negative margins, and adjuvant chemotherapy initiated within 8 weeks of surgery.

Patients with baseline sinus rhythm on 12-lead electrocardiogram (ECG); left ventricular ejection fraction (LVEF) ≥ 55% on transthoracic echocardiography; complete cardiologic examination within 14 days before chemotherapy initiation; and a minimum of 6 months of follow-up data, including ECGs and echocardiographic evaluations at the first and sixth months after chemotherapy initiation, were included in the study.

**Figure 1:**
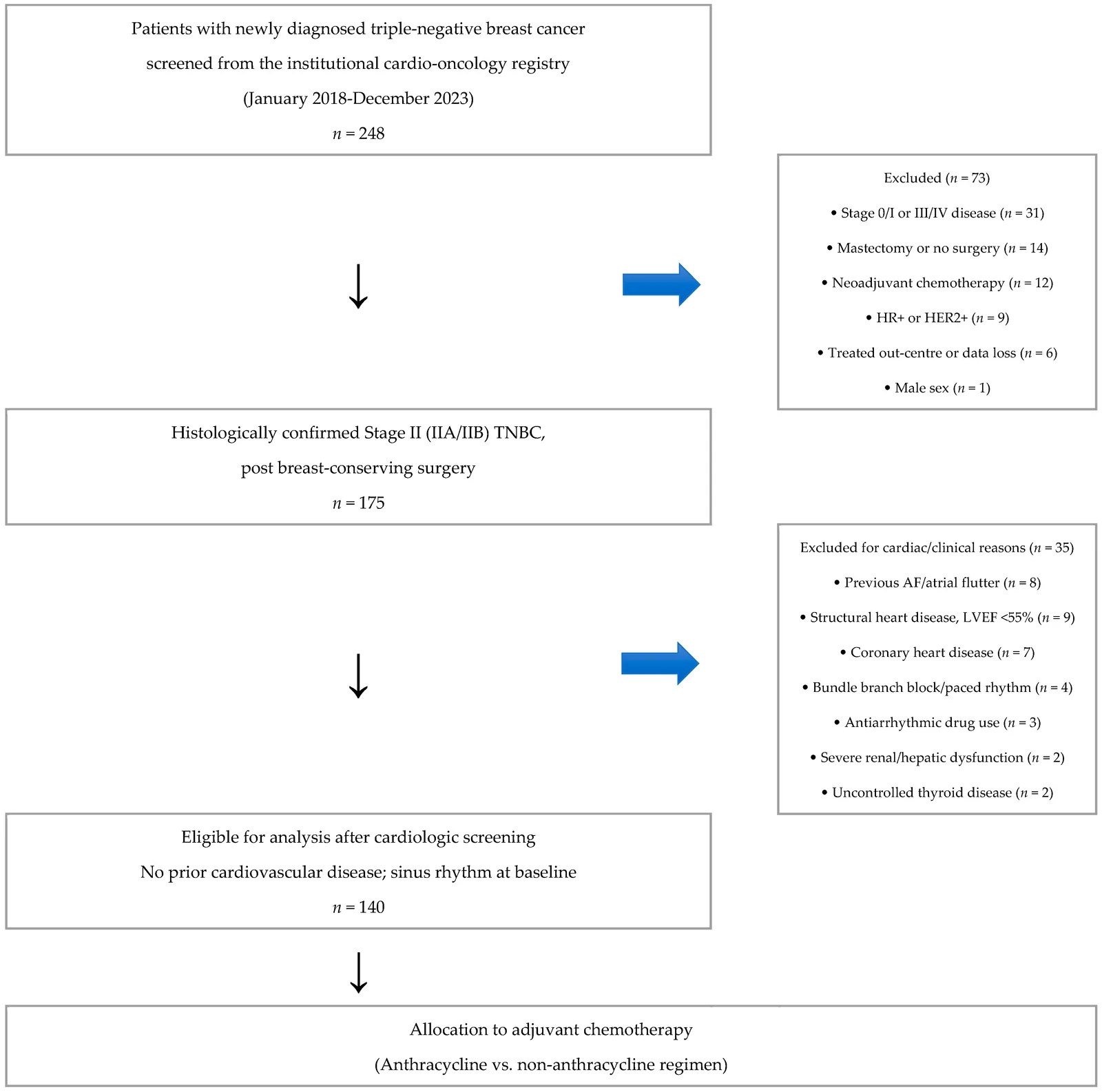

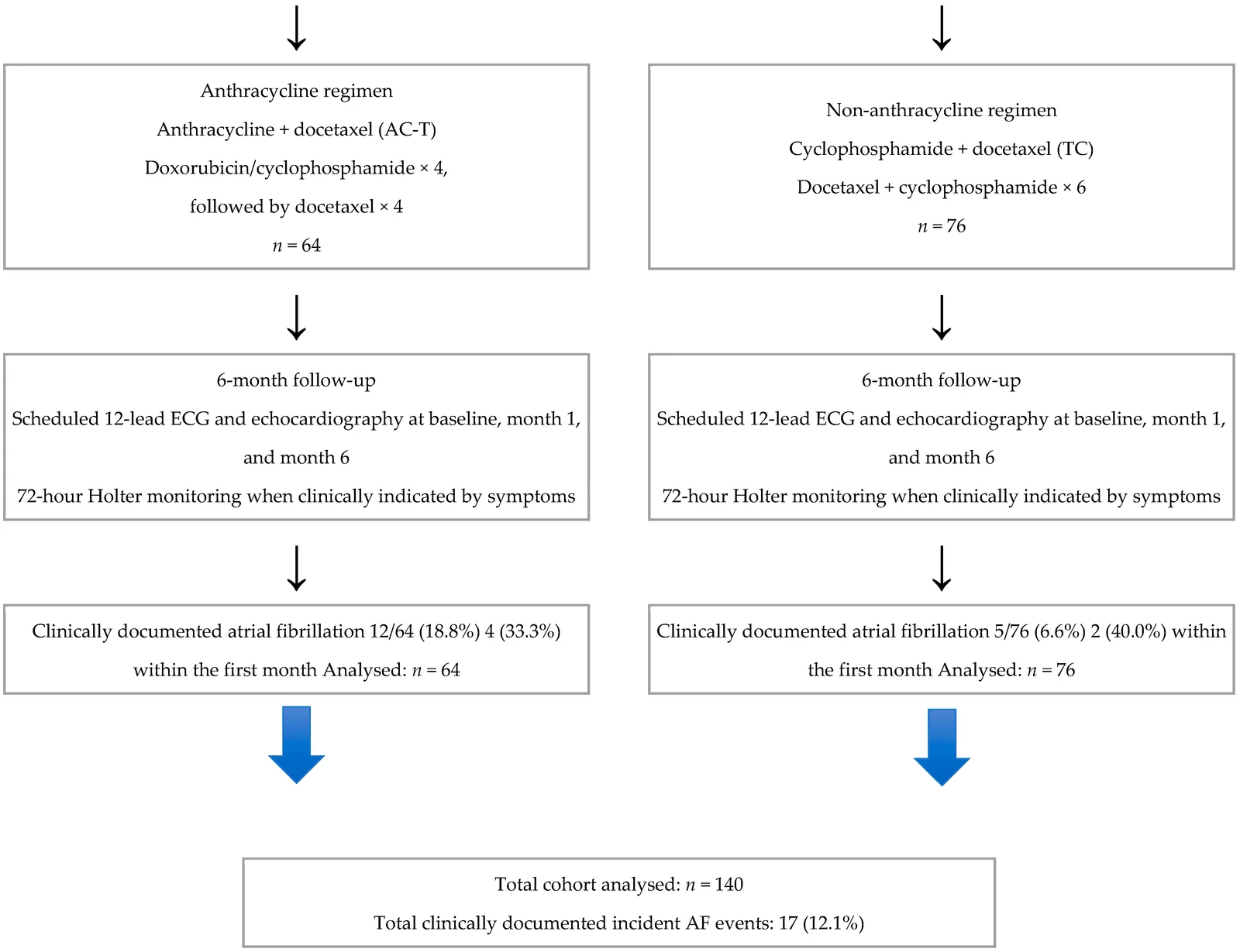
STROBE flow diagram. Abbreviations: AF, atrial fibrillation; ECG, electrocardiography; HER2, human epidermal growth factor receptor 2; HR, hormone receptor; LVEF, left ventricular ejection fraction; TNBC, triple-negative breast cancer.

Exclusion criteria consisted of any non-sinus rhythm, prior history of AF/atrial flutter, sustained supraventricular or ventricular tachyarrhythmia; previously known structural heart disease (moderate-to-severe valvular disease, cardiomyopathy of any aetiology, or congenital heart disease); established coronary heart disease or prior coronary revascularisation; acute coronary syndrome occurring during follow-up; complete left or right bundle branch block, ventricular pre-excitation, or QRS duration ≥ 120 ms on baseline ECG; permanent pacemaker or implantable cardioverter–defibrillator; antiarrhythmic medication use, thyroid dysfunction; estimated glomerular filtration rate < 30 mL/min/1.73 m^2^ or Child-Pugh class B/C hepatic dysfunction; prior mediastinal or left-sided thoracic radiotherapy; previous history of neoadjuvant chemotherapy or any HER2-targeted therapy; concurrent use of investigational agents; any surgical complications prolonging the hospital stay >4 days after breast-conserving surgery, and pregnancy. The study population flow is summarised in Figure 1. We conducted this single-centre retrospective observational cohort study in close collaboration with the Cardiology and Medical Oncology departments of our tertiary centre, and the study was approved by the Institutional Review Board and Ethics Committee (Project No. KA22/151) in accordance with the Declaration of Helsinki. The requirement for written informed consent was waived by the ethics committee. Reporting follows the Strengthening the Reporting of Observational Studies in Epidemiology (STROBE) statement for cohort studies [18].

### 2.2. Therapy Cohort Groups and Exposures

Patients were grouped by adjuvant chemotherapeutic agents. The anthracycline group (AC-T) received 4 cycles of doxorubicin 60 mg/m^2^ + cyclophosphamide 600 mg/m^2^ IV every 21 days, followed by 4 cycles of docetaxel 75 mg/m^2^ every 21 days. The non-anthracycline group (TC) received 6 cycles of docetaxel 75 mg/m^2^ + cyclophosphamide 600 mg/m^2^ every 21 days. The choice of regimen was made by the treating medical oncologist and the patient’s informed preference based on contemporary oncology guidelines [5]. Planned cumulative doses were 240 mg/m^2^ for doxorubicin, 2400 mg/m^2^ for cyclophosphamide, and 300 mg/m^2^ for docetaxel in the AC-T group, and 3600 mg/m^2^ for cyclophosphamide and 450 mg/m^2^ for docetaxel in the TC group. Adjuvant whole-breast radiotherapy was initiated only after completion of chemotherapy and outside the 6-month observation window (Supplementary Table S1).

### 2.3. Cardiologic Assessment

All patients underwent cardiologic physical evaluation at baseline (within 14 days before chemotherapy), in the first month after therapy initiation (month 1), and at month 6 following the start of the chemotherapy regimen. Twelve-lead ECGs were recorded at 25 mm/s and 10 mm/mV in the supine position (Nihon Kohden, Tokyo, Japan) and were retrospectively measured from the digital hospital records by two cardiologists (AA and BH) who were unaware of the chemotherapy regimen and clinical outcomes at the time of measurement. Disagreements were resolved by consensus. Heart rate, PR interval, QRS duration, QT, corrected QT interval (QTc) using Bazett’s formula (QT/√RR), and QT dispersion (QTd = QTmax − QTmin across 12 ECG leads) were recorded. Inter-observer agreement was excellent for all continuous ECG parameters (intraclass correlation coefficients were >0.90).

Transthoracic echocardiography was performed using a GE Vivid-5 system (GE Healthcare, Milwaukee, WI, USA) in accordance with the European Association of Cardiovascular Imaging recommendations [19]. LVEF (modified Simpson’s biplane method), left atrial diameter and indexed volume, transmitral E/A ratio, septal and lateral annular tissue Doppler velocities (E/e′), tricuspid annular plane systolic excursion (TAPSE), and systolic pulmonary artery pressure (sPAP) measurements were obtained retrospectively from the hospital digital records by a staff cardiologist who was unaware of the clinical outcome and chemotherapy allocation of the patient. Ambulatory Holter monitoring (72 h) was performed when clinically indicated by symptoms (palpitations, dyspnoea, presyncope). Holter monitoring was not scheduled universally at fixed study visits. During follow-up, 38 patients underwent at least one symptom-triggered 72 h Holter ECG assessment: 22/64 (34.4%) in the AC-T group and 16/76 (21.1%) in the TC group (*p* = 0.08).

### 2.4. Cardiac Biomarker Assessment

Peripheral venous blood samples were obtained from all participants at baseline (within 24 h prior to the first chemotherapy cycle), at month 1, and at month 6 of follow-up. High-sensitivity cardiac troponin I (hs-cTnI) was analysed using the ARCHITECT STAT High Sensitive Troponin-I assay on the ARCHITECT i2000SR immunoassay analyser (Abbott Diagnostics, Abbott Park, IL, USA) using a chemiluminescent microparticle immunoassay technique. The assay has an analytical limit of detection of 1.7 ng/L, a limit of quantification of 6.0 ng/L at a total coefficient of variation of 10%, and a sex-specific 99th percentile upper reference limit of 16 ng/L for women [20]. Therefore, hs-cTnI ≤ 16 ng/L was considered within the normal range for the present cohort. N-terminal pro-B-type natriuretic peptide (NT-proBNP) was measured using the Elecsys proBNP II electrochemiluminescence immunoassay on the Cobas e601 analyser (Roche Diagnostics GmbH, Mannheim, Germany), with a measuring range of 5–35,000 pg/mL and total imprecision below 5% across the reporting range. In compliance with the 2021 European Society of Cardiology Heart Failure guidelines, NT-proBNP concentrations < 125 pg/mL were accepted to exclude heart failure in ambulatory subjects, with age-adjusted upper reference limits of 450 pg/mL for individuals aged 50–75 years and 900 pg/mL for those >75 years of age [21].

### 2.5. Outcome Definition

The primary outcome was clinically documented incident AF during follow-up, defined according to current European Society of Cardiology guidelines as AF documented on a standard 12-lead ECG of any duration or AF lasting ≥ 30 s on 72 h ambulatory Holter ECG monitoring [1]. Because universal continuous rhythm monitoring was not performed, this endpoint does not represent the total burden of asymptomatic AF. Secondary outcomes included the timing and mode of AF documentation; cancer therapy-related cardiac dysfunction, defined as an absolute LVEF decline of ≥10 percentage points to <50% or new symptomatic heart failure [12]; and the magnitude and direction of ECG interval changes from baseline (Δ; later minus earlier measurement), including ΔPR, ΔQRS, and ΔQTc.

### 2.6. Statistical Analysis

The Shapiro–Wilk test was used to assess normality. Continuous variables are presented as mean ± standard deviation or median (interquartile range), as appropriate, and categorical variables as n (%). Between-group comparisons used the independent-samples t-test, Mann–Whitney U test, Pearson’s chi-square test, or Fisher’s exact test, as appropriate. Patient-level change scores (Δ; later minus earlier measurement) were calculated for baseline-to-month-1, month-1-to-month-6, and baseline-to-month-6 intervals. The cross-sectional between-group comparisons at month 1 and month 6 and, between-group comparisons of patient-level change scores were also analysed. These secondary longitudinal comparisons were exploratory and were not adjusted for multiplicity. Kaplan–Meier analysis and the log-rank test were used to compare clinically documented AF-free survival.

Exploratory univariable logistic regression was used to evaluate candidate clinical and ECG risk markers of clinically documented incident AF. Given the 17 events, no multivariable prognostic model was retained, and these associations are not presented as independent predictors. At month 1, ΔPR > 20 ms was evaluated as a treatment-emergent ECG risk marker rather than as a baseline confounder. To address the six AF events that occurred before the month 1 landmark, a landmark analysis at month 1 was performed, restricted to patients without AF occurring before the month-one landmark, with AF occurring between month 1 and month 6 as the outcome. Models were fitted with and without adjustment for treatment regimen; Firth’s penalised logistic regression with profile-likelihood confidence intervals was used to reduce small-sample bias. The treatment regimen associations were separately evaluated using absolute and relative risks, as well as inverse probability of treatment weighting (IPTW) based only on baseline covariates.

Propensity scores for AC-T treatment were estimated using seven baseline covariates: age, body mass index, AJCC sub-stage, hypertension, diabetes mellitus, LVEF, and QTc; covariate balance was additionally assessed for hyperlipidaemia, smoking, and baseline PR interval. Stabilised inverse probability weights had a mean of 1.01 (range, 0.62–2.41); no truncation was required. Propensity scores demonstrated common support (range, 0.18–0.76), the effective sample size after weighting was 132.8, and all post-weighting standardised mean differences were <0.05 (Supplementary Table S1). The weighted outcome model included only treatment regimen. A two-tailed *p* value < 0.05 was considered significant for the primary regimen comparisons, while all secondary analyses were exploratory. Analyses were performed using IBM SPSS Statistics version 27.0 and R version 4.3.

## 3. Results

### 3.1. Study Population

Of 248 patients screened during the evaluation period, 108 were excluded for stage- or treatment-related reasons (*n* = 73) or cardiac/clinical criteria (*n* = 35), and a final cohort of 140 women was included for analysis (Figure 1). Sixty-four patients (45.7%) received the anthracycline-containing AC-T regimen, and 76 (54.3%) received the non-anthracycline TC regimen. No patient was lost to follow-up. The two groups were well matched for age (64 ± 13 vs. 65 ± 11 years, *p* = 0.77), body mass index, AJCC sub-stage distribution, comorbidity profile, baseline cardiac medications, and baseline cardiac biomarkers (all *p* > 0.10; Table 1). Hypertension was numerically more prevalent in the TC group (44.7% vs. 31.3%, *p* = 0.10) without statistical significance. Baseline HFA-ICOS cardiovascular risk stratification scores were similarly distributed between groups, with most patients classified as ≤moderate risk (81.3% AC-T vs. 81.6% TC, *p* = 0.96). Planned chemotherapy was completed by 61/64 (95.3%) AC-T and 73/76 (96.1%) TC patients (*p* = 1.00); dose reductions occurred in 4/64 (6.3%) and 5/76 (6.6%), respectively (*p* = 1.00). No patient began adjuvant radiotherapy during the six-month observation window (Supplementary Table S1).

### 3.2. Baseline Cardiac Assessment

Baseline echocardiographic and electrocardiographic parameters were comparable between groups (Table 2). Mean baseline LVEF was 57.5 ± 5.6% in the AC-T group and 58.4 ± 4.7% in the TC group (*p* = 0.37), with overall preserved LVEF (58.0 ± 5.1%). Left atrial dimensions, diastolic function indices, and pulmonary artery pressures did not differ between groups. Baseline conduction (PR interval, median [IQR]: 151 [137–165] ms overall; 152 [138–166] ms with AC-T vs. 150 [136–164] ms with TC; *p* = 0.48) and repolarisation (QTc, 423.3 ± 29.0 ms overall) parameters were within reference ranges for women in both groups.

### 3.3. Follow-Up Cardiac Changes

One-month and six-month follow-up data were available for all 140 patients (Table 3). Each *p* value in Table 3 compares AC-T with TC at the corresponding visit and does not represent a within-group or change-from-baseline test. LVEF was comparable at one month (57.7 ± 3.7% vs. 57.7 ± 5.3%; *p* = 0.96) and six months (56.3 ± 6.1% vs. 55.5 ± 5.5%; *p* = 0.42), and no patient met the prespecified definition of cancer therapy-related cardiac dysfunction. At one month, PR distributions were comparable (median [IQR], 162 [148–176] vs. 158 [144–172] ms; *p* = 0.27), whereas hs-cTnI was higher with AC-T (12 [8–17] vs. 9 [5–13] ng/L; *p* = 0.048). At six months, the AC-T group had a higher heart rate (88.5 ± 18.3 vs. 82.9 ± 14.1 bpm; *p* = 0.046), a longer PR interval (160 [148–182] vs. 152 [138–164] ms; *p* = 0.002), and a higher cumulative frequency of clinically documented incident AF (18.8% vs. 6.6%; *p* = 0.037). QRS duration did not differ between groups at six months (*p* = 0.41).

Patient-level longitudinal changes over each prespecified follow-up interval were compared directly between treatment groups (Table 4). From baseline to month 1, PR prolongation was greater with AC-T than with TC (median [IQR], 8.0 [0.0 to 22.0] vs. 2.5 [−4.0 to 11.0] ms; *p* = 0.002), and ΔPR > 20 ms occurred more than three times as frequently (35.9% vs. 10.5%; *p* < 0.001). The early hs-cTnI increase was also greater with AC-T (3.5 [0.5 to 7.2] vs. 1.5 [−1.8 to 4.8] ng/L; *p* = 0.028). From one month to six months, heart rate acceleration was greater with AC-T (+5.9 ± 16.8 vs. +0.7 ± 14.2 bpm; *p* = 0.046), whereas PR change did not differ (*p* = 0.44). From baseline to six months, PR prolongation remained greater with AC-T (6.0 [−2.0 to 20.5] vs. 2.0 [−6.0 to 10.0] ms; *p* = 0.018), and ΔPR > 20 ms remained more frequent (26.6% vs. 10.5%; *p* = 0.013). Conventional echocardiographic changes did not differ significantly. These secondary longitudinal analyses were exploratory.

### 3.4. Incident Atrial Fibrillation

**Figure 2:**
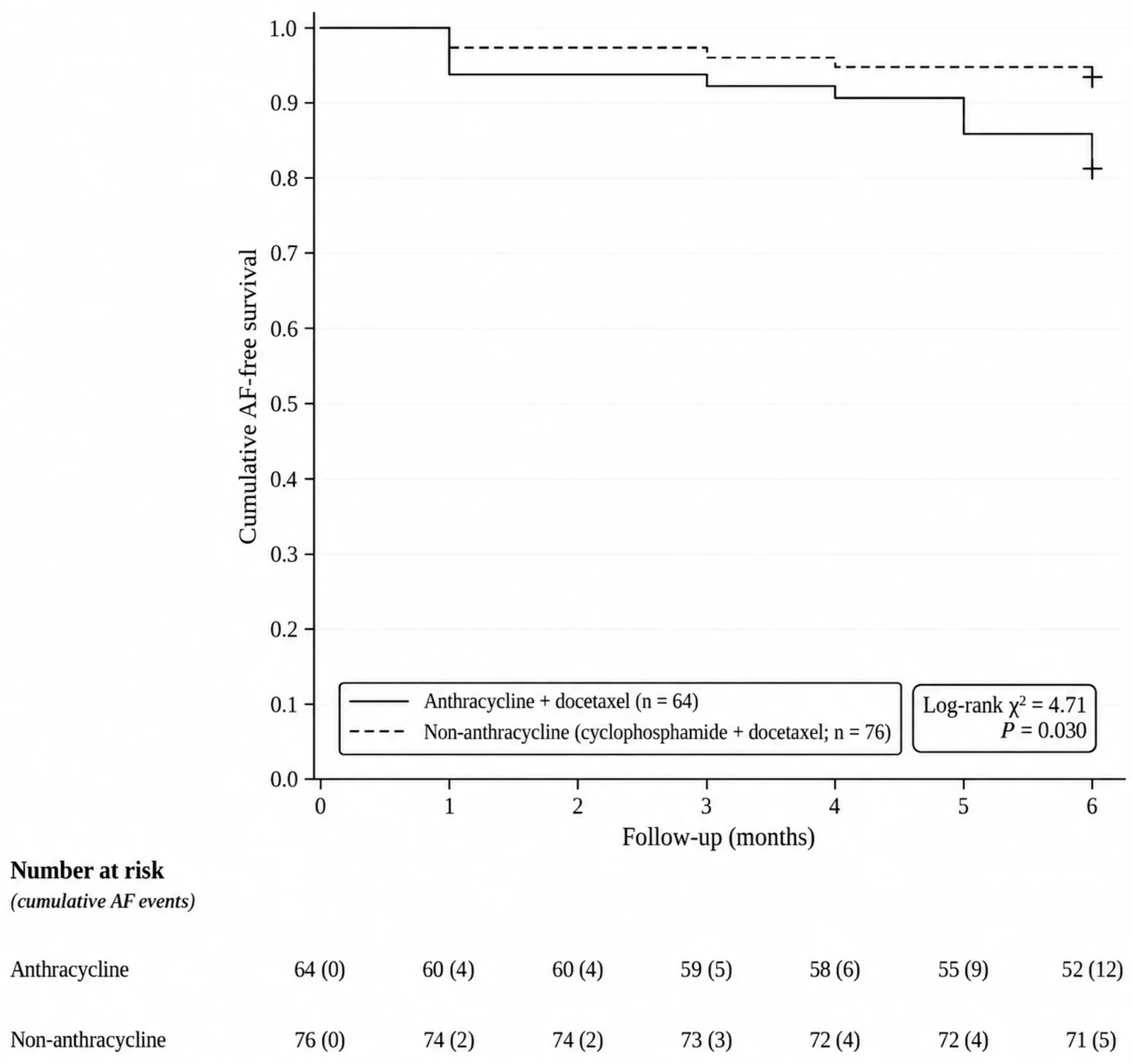
Kaplan–Meier estimates of clinically documented atrial fibrillation-free survival over the 6-month follow-up period, stratified by adjuvant chemotherapy regimen.

Clinically documented incident AF occurred in 17/140 patients (12.1%) during follow-up: 12/64 (18.8%) in the AC-T group and 5/76 (6.6%) in the TC group (*p* = 0.037). Six events occurred within the first month (four AC-T and two TC). Two of these patients (both AC-T) presented in AF that terminated spontaneously, and four were diagnosed by symptom-triggered Holter monitoring or closer follow-up for palpitations; in all six, the one-month ECG used for PR analysis was recorded in sinus rhythm, and no ECG interval was measured during AF. Three of the six (two AC-T, one TC) had ΔPR > 20 ms; because the one-month ECG did not necessarily precede AF in these patients, a landmark analysis was performed (Section 3.5). Thirteen AF cases were documented by 12-lead ECG (four at scheduled visits and nine during unscheduled symptomatic presentations), and four by symptom-triggered 72 h Holter monitoring (AC-T: 3, 6, and 3; TC: 1, 3, and 1, respectively; Supplementary Table S1). Kaplan–Meier curves for clinically documented AF-free survival separated during follow-up (log-rank *p* = 0.030; Figure 2). All episodes were paroxysmal; no thromboembolic event or electrical cardioversion occurred.

### 3.5. Treatment Regimen Association and Exploratory Correlates of Clinically Documented Incident Atrial Fibrillation

Exploratory univariable associations are presented in Table 5. At one month, ΔPR > 20 ms was present in 9/17 patients with clinically documented AF and 22/123 patients without AF (52.9% vs. 17.9%) and was associated with more than fivefold higher odds of the six-month AF endpoint (OR, 5.16; 95% CI, 1.79–14.89; *p* = 0.002). A one-month heart rate increase >10 bpm was also associated with AF (OR, 4.61; 95% CI, 1.59–13.37; *p* = 0.005), as were older age, hypertension, diabetes mellitus, and AC-T treatment. Because six AF events preceded the one-month ECG, the full-cohort estimate is not strictly prospective; ΔPR > 20 ms is therefore regarded as an exploratory treatment-emergent ECG risk marker. In the one-month landmark cohort restricted to patients without AF occurring before the one-month landmark (*n* = 134; AC-T, *n* = 60; TC, *n* = 74; 11 events between month one and month six), AF developed in 6/28 patients (21.4%) with ΔPR > 20 ms versus 5/106 (4.7%) without (Firth OR, 5.33; 95% CI, 1.56–18.94; *p* = 0.008; Table 5). After adjustment for treatment regimen, the association persisted (Firth OR, 3.96; 95% CI, 1.08–15.02; *p* = 0.038), with a consistent direction in both regimens (AC-T, 4/21 vs. 4/39; TC, 2/7 vs. 1/67). No multiplicity adjustment was applied.

The absolute risk difference for clinically documented incident AF with AC-T versus TC was 12.2% (95% CI, 1.1–23.3%), and the unadjusted risk ratio was 2.85 (95% CI, 1.07–7.58). After weighting for baseline covariates, AC-T remained associated with higher odds of clinically documented incident AF (IPTW-adjusted OR, 3.28; 95% CI, 1.05–10.24; *p* = 0.040; Table 6). These estimates describe a regimen-level association and do not isolate the effect of anthracycline exposure from other differences between AC-T and TC.

The AC-T group demonstrated lower clinically documented AF-free survival than the TC group (log-rank χ^2^ = 4.71, *p* = 0.030). Plus signs indicate administrative censoring; the risk table shows patients remaining under observation with cumulative documented AF events in parentheses.

## 4. Discussion

In this single-tertiary-centre retrospective cohort of 140 women with stage II TNBC treated after breast-conserving surgery, the AC-T regimen was associated with a higher frequency of clinically documented incident AF than the TC regimen over six months (18.8% vs. 6.6%; absolute risk difference, 12.2%; risk ratio, 2.85) (Figure 1, Table 3, Table 4, Table 5 and Table 6). The association persisted after IPTW adjustment for measured baseline covariates. At one month, ΔPR > 20 ms was also associated with AF, including in a landmark analysis restricted to patients without AF occurring before the one-month landmark; however, it is a treatment-emergent marker and should not be regarded as an independent or externally validated predictor. Importantly, the PR signal was internally coherent across complementary analyses: AC-T was associated with greater median PR prolongation from baseline to one month (*p* = 0.002), a higher frequency of ΔPR > 20 ms (*p* < 0.001), a longer absolute PR interval at six months (*p* = 0.002), and greater baseline-to-month-six PR prolongation (*p* = 0.018) (Table 3 and Table 4).

The observed AF incidence of 18.8% in anthracycline-treated patients is consistent with the upper range of previously reported rates in heterogeneous breast cancer cohorts, ranging from 2% to 30% [12,15,16]. Anthracycline arrhythmogenicity is currently associated with topoisomerase IIβ inhibition in atrial myocytes, generating DNA double-strand breaks, mitochondrial dysfunction, and reactive oxygen radical accumulation [12,22,23]. Anthracyclines also disrupt proper intracellular calcium handling through ryanodine receptor membrane leak and sarcoplasmic reticulum calcium ATPase impairment, eventually predisposing to triggered atrial activity [13,23]. Furthermore, multiple cardiac ion channel modulation (including hERG, sodium, and L-type calcium channels) may also lead to heterogeneous repolarisation and conduction slowing, which may precipitate re-entrant arrhythmias [24]. Our finding that one-month PR prolongation was associated with clinically documented AF, together with a nonsignificant trend toward slightly longer QRS durations and QTc intervals in the anthracycline arm during follow-up, is consistent with these mechanistic observations regarding alterations in both depolarization and repolarization features of the cardiac tissue. Subclinical early involvement of the atrial conduction characteristics may serve as a surrogate marker of an arrhythmogenic substrate before a manifest rhythm disturbance [23,24]. Supporting this hypothesis, Sandamali et al. [25] prospectively examined ECG parameters of breast cancer patients before, during, and six months after anthracycline chemotherapy and observed a significant increase in the PR interval six months after the completion of therapy without the PR interval reaching values indicative of first-degree heart block. Yaylali et al. [26] further demonstrated a significant prolongation of atrial electromechanical delay, which is a well-established surrogate marker for incident AF, in anthracycline-treated breast cancer patients, suggesting that subclinical adverse electrical remodelling may occur earlier, providing an arrhythmogenic substrate prior to the manifestation of overt clinical arrhythmias. Similarly, in another contemporary study [27], anthracycline-induced ventricular repolarization abnormalities (JT-corrected and QTc-dispersion) were shown to significantly precede overt systolic chemotherapy-related cardiac dysfunction and emerged as early as the second chemotherapy cycle.

In our cohort, heart rates also significantly increased in the AC-T group at the end of the six-month follow-up period, aligning with the previously stated acute chronotropic effects of anthracycline regimens, as detailed in recent meta-analyses and electrocardiographic studies [15,16,25]. According to the current cardio-oncology guidelines, these early alterations in heart rate might be an indicator of acute cardiotoxicity that should be taken into consideration [12]. Furthermore, this probable early acute myocardial stress might also be reflected in our biomarker analysis. We observed a borderline statistically significant elevation in hs-Troponin I during the first month in the AC-T group, which no longer differed between groups at six months. This early transient troponin release is, in fact, a previously known phenomenon reflecting acute myocardial cell injury following immediate anthracycline exposure, rather than permanent structural damage in the initial stage [28,29,30,31]. In contrast, NT-proBNP levels remained slightly elevated from baseline levels without statistical significance across follow-up points in both groups but more evidently in the AC-T arm (Table 3). As discussed in the current literature, this discrepancy between troponin and BNP levels suggests that while acute cellular injury (indicated by hs-TnI) occurs early, significant myocardial stretch, which would be required for a marked NT-proBNP elevation, has not yet developed in this initial phase [12,30]. These biomarker comparisons were secondary and exploratory.

However, anthracyclines may elicit subclinical myocardial stress detectable by biomarker assays before overt cardiac dysfunction develops [28,29]. In our cohort, no patient met the prespecified definition of cancer therapy-related cardiac dysfunction. These observations suggest that electrical and arrhythmic changes may precede overt systolic impairment.

From a practical perspective, our findings may support a more careful on-treatment ECG assessment during anthracycline-containing chemotherapy, with particular attention to PR interval prolongation, even in the normal PR interval range, but they cannot establish an actionable threshold for Holter or wearable monitoring. QRS duration, NT-proBNP, and conventional echocardiographic changes did not differ significantly between regimens. The finding of ΔPR > 20 ms in the first month of the AC-T regimen should therefore be viewed as a clinically interesting, hypothesis-generating ECG signal.

The 2022 ESC Guidelines on cardio-oncology recommend baseline and on-treatment ECG assessment in patients receiving cardiotoxic chemotherapy [12] but do not specify a PR-based surveillance threshold. Our data generate the hypothesis that one-month PR prolongation may refine arrhythmic risk assessment; prospective multicentre studies with systematic rhythm monitoring are required before this observation can alter clinical surveillance pathways.

### Study Limitations

Several limitations should be addressed. First, our single-centre retrospective design may have introduced selection bias. Although consecutive enrolment and IPTW adjustment improved comparability between the groups, residual confounding due to unmeasured factors cannot be excluded. Second, treatment allocation was not randomised, introducing possible confounding by indication. AC-T and TC are complete regimens that differ in composition, sequence, cycle number, and duration; therefore, the association cannot be attributed exclusively to anthracycline. Third, the modest number of AF events (*n* = 17) precluded a stable multivariable prognostic model; the univariable risk marker analyses are exploratory. Fourth, rhythm monitoring relied on scheduled or symptom-triggered 12-lead ECGs and symptom-triggered 72 h Holter monitoring. Asymptomatic or short AF episodes may therefore have been missed. In addition, six AF events preceded the one-month ECG; although the association of ΔPR > 20 ms with AF was preserved in a landmark analysis restricted to patients without AF occurring before the one-month landmark, the number of events in this analysis was small (*n* = 11). Fifth, the six-month follow-up did not permit assessment of longer-term AF risk. Sixth, strain echocardiography was unavailable, potentially limiting the precision of our guideline-directed [12] cancer therapy-related cardiac dysfunction results. Seventh, the longitudinal delta analyses included multiple secondary comparisons without multiplicity adjustment and remain exploratory. Finally, the single-centre cohort of middle-aged Turkish women limits generalisability. Validation in larger prospective multicentre cohorts with systematic rhythm monitoring and longer follow-up is warranted.

## 5. Conclusions

In women with stage II TNBC treated after breast-conserving surgery, the AC-T regimen was associated with a higher frequency of clinically documented incident AF than the TC regimen over six months. The absolute risk difference was 12.2%, and the unadjusted risk ratio was 2.85; however, the retrospective non-randomised design precludes causal attribution to anthracycline alone. Early PR prolongation, particularly ΔPR > 20 ms, was associated with subsequent AF in exploratory analyses, including a landmark analysis, and represents a candidate treatment-emergent ECG risk marker. Prospective multicentre studies with systematic long-term rhythm monitoring should determine whether this signal can be validated and translated into a monitoring threshold for anthracycline-containing chemotherapy regimens.

## Figures and Tables

**Table 1:** Baseline demographic and clinical characteristics of the study population.

Characteristic	Anthracycline + Docetaxel (*n* = 64)	Cyclophosphamide + Docetaxel (*n* = 76)	Overall (*n* = 140)	*p*
Age, y, mean ± SD	64 ± 13	65 ± 11	64 ± 12	0.77
Body mass index, kg/m^2^, mean ± SD	27.4 ± 2.6	28.1 ± 3.5	27.7 ± 3.2	0.31
AJCC stage IIA, *n* (%)	30 (46.9)	37 (48.7)	67 (47.9)	0.83
AJCC stage IIB, *n* (%)	34 (53.1)	39 (51.3)	73 (52.1)	0.83
Hypertension, *n* (%)	20 (31.3)	34 (44.7)	54 (38.6)	0.10
Diabetes mellitus, *n* (%)	14 (21.9)	14 (18.4)	28 (20.0)	0.61
Hyperlipidaemia, *n* (%)	16 (25.0)	21 (27.6)	37 (26.4)	0.73
Current smoker, *n* (%)	17 (26.6)	15 (19.7)	32 (22.9)	0.34
Family history of CVD, *n* (%)	11 (17.2)	13 (17.1)	24 (17.1)	0.99
HFA-ICOS baseline risk ≤ moderate, *n* (%)	52 (81.3)	62 (81.6)	114 (81.4)	0.96
Concomitant cardiac medications				
Beta-blocker, *n* (%)	8 (12.5)	11 (14.5)	19 (13.6)	0.74
ACE-i/ARB, *n* (%)	13 (20.3)	20 (26.3)	33 (23.6)	0.41
Calcium channel blocker, *n* (%)	5 (7.8)	7 (9.2)	12 (8.6)	0.77
Statin, *n* (%)	9 (14.1)	11 (14.5)	20 (14.3)	0.95
Baseline cardiac biomarkers				
NT-proBNP, pg/mL, median (IQR)	109 (78–168)	106 (72–155)	107 (74–161)	0.87
hs-Troponin I, ng/L, median (IQR)	8 (5–12)	7 (5–11)	8 (5–12)	0.62

Values are presented as mean ± standard deviation, median (interquartile range), or *n* (%). ACE-i, angiotensin-converting enzyme inhibitor; AJCC, American Joint Committee on Cancer; ARB, angiotensin II receptor blocker; CVD, cardiovascular disease; hs, high-sensitivity; IQR, interquartile range; NT-proBNP, N-terminal pro-B-type natriuretic peptide. *p* values were calculated using the independent samples *t*-test, Mann–Whitney U test, Pearson χ^2^, or Fisher’s exact test as appropriate.

**Table 2:** Baseline echocardiographic and electrocardiographic parameters by chemotherapy group.

Characteristic	Anthracycline + Docetaxel (AC-T, *n* = 64)	Cyclophosphamide + Docetaxel (TC, *n* = 76)	Overall (*n* = 140)	*p* Value
Echocardiographic variables				
LVEF, %, mean ± SD	57.5 ± 5.6	58.4 ± 4.7	58.0 ± 5.1	0.37
LA diameter, mm, median (IQR)	37.0 (34.0–40.0)	36.5 (33.5–39.8)	36.8 (33.8–39.9)	0.78
LA volume index, mL/m^2^, mean ± SD	27.4 ± 5.3	26.8 ± 5.9	27.1 ± 5.6	0.54
E/e′ ratio, mean ± SD	7.9 ± 2.4	7.6 ± 2.4	7.7 ± 2.4	0.55
Diastolic dysfunction, n (%)	38 (59.4)	38 (50.0)	76 (54.3)	0.31
sPAP, mmHg, mean ± SD	29.2 ± 6.8	30.6 ± 6.7	30.0 ± 6.7	0.43
TAPSE, mm, median (IQR)	21.0 (19.8–22.5)	21.0 (19.8–22.4)	21.0 (19.8–22.5)	0.97
Electrocardiographic variables				
Heart rate, bpm, mean ± SD	81.4 ± 16.5	79.8 ± 12.3	80.4 ± 14.5	0.58
PR interval, ms, median (IQR)	152 (138–166)	150 (136–164)	151 (137–165)	0.48
P-wave duration, ms, mean ± SD	104.2 ± 11.8	103.6 ± 10.9	103.9 ± 11.3	0.75
QRS duration, ms, mean ± SD	91.3 ± 17.0	92.2 ± 14.0	91.8 ± 15.4	0.74
QTc interval (Bazett), ms, mean ± SD	425.0 ± 31.4	421.9 ± 27.1	423.3 ± 29.0	0.53
QT dispersion, ms, mean ± SD	38.9 ± 9.7	37.8 ± 8.4	38.3 ± 9.0	0.47

Values are presented as mean ± SD, median (interquartile range [IQR]: 25th–75th percentiles), or *n* (%), as appropriate. *p* values were calculated using the independent-samples t-test for normally distributed continuous variables, the Mann–Whitney U test for non-normally distributed continuous variables (PR interval, LA diameter, and TAPSE), and Pearson’s chi-square test for categorical variables. AC-T, anthracycline plus docetaxel; LA, left atrium; LVEF, left ventricular ejection fraction; QTc, corrected QT interval (Bazett); SD, standard deviation; sPAP, systolic pulmonary artery pressure; TAPSE, tricuspid annular plane systolic excursion; TC, cyclophosphamide plus docetaxel.

**Table 3:** Echocardiographic, electrocardiographic, biomarker, and clinical parameters at month 1 and month 6 of follow-up.

Parameter	Month 1			Month 6		
	AC-T (*n* = 64)	TC (*n* = 76)	Between-Group *p* Value	AC-T (*n* = 64)	TC (*n* = 76)	Between-Group *p* Value
Echocardiographic						
LVEF, %, mean ± SD	57.7 ± 3.7	57.7 ± 5.3	0.96	56.3 ± 6.1	55.5 ± 5.5	0.42
LA diameter, mm, median (IQR)	37.8 (35.5–40.0)	36.5 (34.0–39.2)	0.06	38.0 (35.2–40.4)	38.2 (34.8–41.5)	0.79
E/e′ ratio, mean ± SD	7.8 ± 1.6	8.1 ± 2.3	0.45	8.4 ± 1.5	8.1 ± 2.3	0.36
sPAP, mmHg, mean ± SD	31.1 ± 6.9	31.8 ± 5.6	0.70	32.5 ± 4.2	33.7 ± 11.0	0.38
TAPSE, mm, median (IQR)	20.8 (19.2–22.0)	21.0 (19.5–22.2)	0.44	21.2 (19.8–22.5)	20.2 (19.0–21.0)	0.14
Diastolic dysfunction, *n* (%)	39 (60.9)	43 (56.6)	0.59	44 (68.8)	50 (65.8)	0.73
Electrocardiographic						
Heart rate, bpm, mean ± SD	82.6 ± 15.5	82.2 ± 16.4	0.89	88.5 ± 18.3	82.9 ± 14.1	**0.046**
PR interval, ms, median (IQR)	162 (148–176)	158 (144–172)	0.27	160 (148–182)	152 (138–164)	**0.002**
QRS duration, ms, mean ± SD	90.6 ± 10.9	88.3 ± 11.1	0.22	95.4 ± 16.7	93.3 ± 12.5	0.41
QTc interval (Bazett), ms, mean ± SD	435.2 ± 36.0	431.3 ± 46.8	0.59	443.2 ± 36.3	437.7 ± 52.1	0.48
Cardiac biomarkers						
NT-proBNP, pg/mL, median (IQR)	185 (112–340)	142 (88–226)	0.16	214 (98–365)	152 (92–240)	0.49
hs-cTnI, ng/L, median (IQR)	12 (8–17)	9 (5–13)	**0.048**	10 (6–15)	8 (5–12)	0.28
Clinically documented AF, *n* (%)	4 (6.3)	2 (2.6)	0.41	12 (18.8)	5 (6.6)	**0.037**

Values are presented as mean ± SD, median (interquartile range [IQR]: 25th–75th percentiles), or *n* (%), as appropriate. *p* values represent cross-sectional between-group comparisons at month 1 and month 6 using independent-samples *t*-tests for normally distributed continuous variables, the Mann–Whitney U test for non-normally distributed continuous variables (PR interval, NT-proBNP, hs-cTnI, LA diameter, and TAPSE), and Pearson’s chi-square test or Fisher’s exact test for categorical variables. Statistically significant *p* values (<0.05) are shown in bold. AC-T, anthracycline plus docetaxel; AF, atrial fibrillation; hs-cTnI, high-sensitivity cardiac troponin I; LA, left atrium; LVEF, left ventricular ejection fraction; NT-proBNP, N-terminal pro-B-type natriuretic peptide; QTc, corrected QT interval; SD, standard deviation; sPAP, systolic pulmonary artery pressure; TAPSE, tricuspid annular plane systolic excursion; TC, cyclophosphamide plus docetaxel.

**Table 4:** Longitudinal patient-level changes (Δ) in echocardiographic, electrocardiographic, and biomarker parameters between treatment groups.

Parameter	AC-T (*n* = 64)	TC (*n* = 76)	Between-Group *p* Value
A. Changes from baseline to month 1 (ΔM1-Baseline)			
ΔVEF, %, mean ± SD	+0.2 ± 4.8	−0.7 ± 5.1	0.29
ΔLA diameter, mm, median (IQR)	0.6 (−1.8 to 3.1)	−0.2 (−3.4 to 2.9)	0.11
ΔTAPSE, mm, median (IQR)	−0.4 (−1.7 to 0.9)	−0.2 (−1.4 to 1.0)	0.38
ΔHeart rate, bpm, mean ± SD	+1.2 ± 15.8	+2.4 ± 14.6	0.64
ΔPR interval, ms, median (IQR)	8.0 (0.0 to 22.0)	2.5 (−4.0 to 11.0)	**0.002**
Patients with ΔPR > 20 ms, *n* (%)	23 (35.9%)	8 (10.5%)	**<0.001**
ΔQRS duration, ms, mean ± SD	−0.7 ± 14.5	−3.9 ± 13.2	0.17
ΔQTc interval, ms, mean ± SD	+10.2 ± 33.4	+9.4 ± 38.6	0.89
Δhs-cTnI, ng/L, median (IQR)	3.5 (0.5 to 7.2)	1.5 (−1.8 to 4.8)	**0.028**
ΔNT-proBNP, pg/mL, median (IQR)	72 (−15 to 186)	36 (−18 to 92)	0.12
B. Changes from month 1 to month 6 (ΔM6-M1)			
Δ LVEF, %, mean ± SD	−1.4 ± 5.1	−2.2 ± 4.8	0.34
Δ LA diameter, mm, median (IQR)	0.0 (−1.8 to 1.7)	1.8 (−0.6 to 4.0)	0.08
Δ E/e′ ratio, mean ± SD	+0.6 ± 1.8	+0.0 ± 2.1	0.22
Δ sPAP, mmHg, mean ± SD	+1.4 ± 6.2	+1.9 ± 8.4	0.69
Δ TAPSE, mm, median (IQR)	0.5 (−1.0 to 1.9)	−0.8 (−2.1 to 0.4)	0.11
Δ Heart rate, bpm, mean ± SD	+5.9 ± 16.8	+0.7 ± 14.2	**0.046**
Δ PR interval, ms, median (IQR)	0.0 (−10.0 to 8.0)	0.0 (−8.0 to 6.0)	0.44
Patients with ΔPR > 20 ms, *n* (%)	5 (7.8%)	3 (3.9%)	0.32
Δ QRS duration, ms, mean ± SD	+4.8 ± 14.6	+5.0 ± 11.8	0.93
Δ QTc interval, ms, mean ± SD	+8.0 ± 32.5	+6.4 ± 44.1	0.81
Δ hs-cTnI, ng/L, median (IQR)	−1.0 (−4.8 to 2.8)	−1.0 (−4.4 to 2.4)	0.92
Δ NT-proBNP, pg/mL, median (IQR)	−45 (−138 to 32)	12 (−19 to 46)	0.28
C. Overall changes from baseline to month 6 (ΔM6-Baseline)			
Δ LVEF, %, mean ± SD	−1.2 ± 5.9	−2.9 ± 5.3	0.08
Δ LA diameter, mm, median (IQR)	0.6 (−2.1 to 3.4)	1.4 (−1.9 to 5.1)	0.33
Δ E/e′ ratio, mean ± SD	+0.5 ± 2.0	+0.5 ± 2.4	1.00
Δ sPAP, mmHg, mean ± SD	+3.3 ± 6.6	+3.1 ± 8.9	0.88
Δ TAPSE, mm, median (IQR)	0.0 (−1.4 to 1.4)	−1.0 (−2.3 to 0.3)	0.14
Δ Heart rate, bpm, mean ± SD	+7.1 ± 17.5	+3.1 ± 13.5	0.13
Δ PR interval, ms, median (IQR)	6.0 (−2.0 to 20.5)	2.0 (−6.0 to 10.0)	**0.018**
Patients with ΔPR > 20 ms, *n* (%)	17 (26.6%)	8 (10.5%)	**0.013**
Δ QRS duration, ms, mean ± SD	+4.1 ± 17.1	+1.1 ± 13.4	0.24
Δ QTc interval, ms, mean ± SD	+18.2 ± 34.1	+15.8 ± 42.0	0.71
Δ hs-cTnI, ng/L, median (IQR)	2.5 (−1.4 to 6.6)	1.0 (−2.2 to 4.2)	0.11
Δ NT-proBNP, pg/mL, median (IQR)	105 (−8 to 210)	46 (12 to 80)	0.37

Values are presented as mean ± SD, median (interquartile range [IQR]: 25th–75th percentiles), or *n* (%), as appropriate. Δ represents the intra-individual difference calculated as the later measurement minus the earlier measurement. Continuous change scores were compared using independent-samples *t*-tests for normally distributed variables and Mann–Whitney U tests for non-normally distributed variables (ΔPR interval, Δhs-cTnI, ΔNT-proBNP, ΔLA diameter, and ΔTAPSE). The month-1-to-month-6 ΔE/e′ comparison was also performed using the Mann–Whitney U test because its change-score distribution was non-normally distributed; its descriptive values are retained as mean ± SD for consistency across echocardiographic intervals. Categorical variables, including ΔPR > 20 ms, were compared using Pearson’s chi-square test or Fisher’s exact test. Statistically significant *p* values (<0.05) are shown in bold. These secondary longitudinal comparisons were exploratory and were not adjusted for multiplicity. AC-T, anthracycline plus docetaxel; hs-cTnI, high-sensitivity cardiac troponin I; LA, left atrium; LVEF, left ventricular ejection fraction; NT-proBNP, N-terminal pro-B-type natriuretic peptide; QTc, corrected QT interval; SD, standard deviation; sPAP, systolic pulmonary artery pressure; TAPSE, tricuspid annular plane systolic excursion; TC, cyclophosphamide plus docetaxel.

**Table 5:** Exploratory univariable associations with clinically documented incident atrial fibrillation at 6-month follow-up.

Variable (Exploratory)	AF (*n* = 17)	No AF (*n* = 123)	OR (95% CI)	*p*
Age, per 1-y increase	70 ± 10	63 ± 12	1.06 (1.01–1.11)	**0.012**
Age ≥ 65 years, *n* (%)	13 (76.5)	60 (48.8)	3.41 (1.07–10.85)	**0.038**
Body mass index, per 1 kg/m^2^	28.0 ± 3.4	27.6 ± 3.1	1.04 (0.91–1.19)	0.55
Hypertension, *n* (%)	12 (70.6)	42 (34.1)	4.63 (1.55–13.82)	**0.006**
Diabetes mellitus, *n* (%)	7 (41.2)	21 (17.1)	3.40 (1.16–10.00)	**0.026**
Hyperlipidaemia, *n* (%)	5 (29.4)	32 (26.0)	1.18 (0.39–3.62)	0.77
Current smoker, *n* (%)	4 (23.5)	28 (22.8)	1.05 (0.32–3.44)	0.94
AJCC stage IIB, *n* (%)	10 (58.8)	63 (51.2)	1.36 (0.49–3.78)	0.56
HFA-ICOS baseline risk ≤ moderate, *n* (%)	14 (82.4)	100 (81.3)	1.08 (0.30–3.86)	0.91
AC-T regimen, *n* (%)	12 (70.6)	52 (42.3)	3.28 (1.09–9.87)	**0.037**
Baseline LVEF, per 1%	57.4 ± 5.0	58.1 ± 5.1	0.97 (0.89–1.07)	0.55
Baseline LA diameter, mm	38.3 ± 5.6	36.7 ± 5.0	1.07 (0.97–1.18)	0.18
Baseline QTc, ms	429 ± 32	423 ± 28	1.01 (0.99–1.03)	0.32
Δ Heart rate > 10 bpm at month 1	11 (64.7)	35 (28.5)	4.61 (1.59–13.37)	**0.005**
Month-1 ΔPR > 20 ms	9 (52.9)	22 (17.9)	5.16 (1.79–14.89)	**0.002**
Month-1 ΔPR > 20 ms, landmark cohort †	6/11 subsequent AF (54.5)	22/123 no subsequent AF (17.9)	5.33 (1.56–18.94)	**0.008**
Δ QTc > 50 ms at month 1	6 (35.3)	20 (16.3)	2.81 (0.93–8.46)	0.066

CI, confidence interval; LA, left atrium; LVEF, left ventricular ejection fraction; OR, odds ratio; QTc, corrected QT interval. Δ indicates change from baseline to month 1. Month-1 change variables were evaluated as treatment-emergent ECG risk markers. Because six AF events preceded the month-1 ECG, the full-cohort ΔPR estimate is not strictly prospective; a landmark analysis restricted to patients without AF occurring before the month-1 landmark is therefore also shown (†). No multiplicity adjustment was applied; *p* values are exploratory. † Landmark analysis restricted to patients without AF occurring before the month-1 landmark (*n* = 134; 11 subsequent AF events between month 1 and month 6); Firth penalised OR with profile-likelihood 95% CI. After adjustment for treatment regimen: OR, 3.96 (95% CI, 1.08–15.02; *p* = 0.038). Statistically significant *p* values (<0.05) are shown in bold.

**Table 6:** Absolute, relative, and IPTW-adjusted measures of the association between chemotherapy regimen and clinically documented incident atrial fibrillation.

Effect Measure	Estimate	95% CI	*p*
Absolute risk difference, AC-T vs. TC	12.2%	1.1–23.3%	0.037
Unadjusted risk ratio, AC-T vs. TC	2.85	1.07–7.58	0.037
IPTW-adjusted odds ratio, AC-T vs. TC	3.28	1.05–10.24	0.040

CI, confidence interval; IPTW, inverse probability of treatment weighting. IPTW used baseline covariates only; full weighting diagnostics and balance measures are reported in Supplementary Table S1.

## Data Availability

The data supporting the findings of this study are not publicly available due to patient privacy and ethical restrictions. The data may be made available by the corresponding author upon reasonable request and with appropriate justification, subject to applicable ethical and institutional requirements.

## Supplementary Materials

The following supporting information can be downloaded at: https://www.mdpi.com/article/10.3390/biomedicines14102214/s1, Table S1: IPTW Diagnostics, Covariate Balance, Treatment Delivery, and Rhythm-Monitoring Characteristics.

## Abbreviations

The following abbreviations are used in this manuscript:

AF	Atrial fibrillation
TNBC	Triple-negative breast cancer
AC-T	Anthracycline + Docetaxel
TC	Cyclophosphamide + Docetaxel
HFA-ICOS	Heart Failure Association-International Cardio-Oncology Society
AJCC	American Joint Committee on Cancer
