# Supplementary material for: Incident Atrial Fibrillation Following Anthracycline-Based Adjuvant Chemotherapy in Women with Breast Cancer: A Single-Centre Retrospective Cohort Study
Source: Biomedicines. 2026 Sep 30;14(10):2214. doi: 10.3390/biomedicines14102214 (PMC13701210; doi:10.3390/biomedicines14102214)
Supplement: Supplementary file 1 [file biomedicines-14-02214-s001.zip › biomedicines-4581605-supplementary.pdf]

Supplementary Table S1. IPTW Diagnostics, Covariate Balance, Treatment Delivery, and Rhythm-Monitoring Characteristics

A. Baseline Covariate Balance Before and After Stabilised IPTW

Baseline covariate	Unweighted AC-T (n = 64)	Unweighted TC (n = 76)	SMD before	IPTW AC-T (effective n = 61.2)	IPTW TC (effective n = 71.6)	SMD after
Age, years, mean ± SD	64.0 ± 13.0	65.0 ± 11.0	0.083	64.6 ± 12.1	64.4 ± 11.8	0.017
Body mass index, kg/m², mean ± SD	27.4 ± 2.6	28.1 ± 3.5	0.226	27.8 ± 3.1	27.7 ± 3.2	0.032
AJCC stage IIB, n (%)	34 (53.1)	39 (51.3)	0.036	52.0%	52.4%	0.008
Hypertension, n (%)	20 (31.3)	34 (44.7)	0.280	38.2%	38.9%	0.014
Diabetes mellitus, n (%)	14 (21.9)	14 (18.4)	0.087	20.1%	19.8%	0.007
Hyperlipidaemia, n (%) *	16 (25.0)	21 (27.6)	0.060	26.2%	26.5%	0.007
Current smoker, n (%) *	17 (26.6)	15 (19.7)	0.163	23.1%	22.8%	0.007
Baseline LVEF, %, mean ± SD	57.5 ± 5.6	58.4 ± 4.7	0.174	58.0 ± 5.1	57.9 ± 4.9	0.020
Baseline QTc, ms, mean ± SD	425.0 ± 31.4	421.9 ± 27.1	0.106	423.5 ± 28.9	423.1 ± 28.4	0.014
Baseline PR interval, ms, mean ± SD *	153.8 ± 24.3	150.7 ± 21.2	0.136	152.1 ± 22.8	152.3 ± 22.0	0.009

Propensity-score range, 0.18-0.76; stabilised weight mean, 1.01 (range, 0.62-2.41); no truncation; total effective sample size, 132.8. SMD <0.10 indicates adequate balance. The propensity score was estimated from age, body mass index, AJCC sub-stage, hypertension, diabetes mellitus, baseline LVEF, and baseline QTc. * Not included in the propensity score model; balance assessed descriptively. Weighted categorical variables are presented as percentages.

B. Adjuvant Chemotherapy Exposure and Delivery

Characteristic	AC-T (n = 64)	TC (n = 76)	P
Cumulative doxorubicin dose, mg/m², median (IQR)	240 (240-240)	—	—
Cumulative cyclophosphamide dose, mg/m², median (IQR)	2400 (2400-2400)	3600 (3600-3600)	—
Cumulative docetaxel dose, mg/m², median (IQR)	300 (300-300)	450 (450-450)	—
Full planned chemotherapy completion, n (%)	61 (95.3)	73 (96.1)	1.00
Dose reduction in ≥1 cycle, n (%)	4 (6.3)	5 (6.6)	1.00
Treatment-cycle delay >7 days, n (%)	6 (9.4)	7 (9.2)	1.00
Discontinuation due to toxicity, n (%)	3 (4.7)	3 (3.9)	1.00
Radiotherapy initiated during 6-month window, n (%)	0 (0.0)	0 (0.0)	—
Timing of whole-breast radiotherapy	After chemotherapy (>6 months)	After chemotherapy (>6 months)	—

C. Rhythm-Monitoring and AF-Ascertainment Characteristics

Characteristic	AC-T	TC	Overall / AF cases	P
Patients undergoing ≥1 symptom-triggered 72-hour Holter	22/64 (34.4%)	16/76 (21.1%)	38/140 (27.1%)	0.08
AF documented by scheduled 12-lead ECG	3	1	4/17 (23.5%)	—
AF documented by unscheduled symptomatic 12-lead ECG	6	3	9/17 (52.9%)	—
AF documented by symptom-triggered 72-hour Holter	3	1	4/17 (23.5%)	—

Values are mean ± SD, median (IQR), or n (%) as indicated. Categorical comparisons in panel B used Fisher's exact test; the Holter comparison in panel C used Pearson's chi-square test. AF, atrial fibrillation; AC-T, doxorubicin plus cyclophosphamide followed by docetaxel; AJCC, American Joint Committee on Cancer; IPTW, inverse probability of treatment weighting; LVEF, left ventricular ejection fraction; SMD, standardised mean difference; TC, cyclophosphamide + docetaxel. Holter monitoring was symptom-triggered and was not scheduled at fixed study time points. Whole-breast radiotherapy began only after chemotherapy and outside the 6-month cardiac follow-up window. For IPTW balance diagnostics, continuous covariates are summarised using weighted means and standard deviations for SMD calculation; this diagnostic presentation does not replace the distribution-appropriate median (IQR) reporting and non-parametric inference for PR interval in main-text Tables 2-4.
